# Determination of a six-gene prognostic model for cervical cancer based on WGCNA combined with LASSO and Cox-PH analysis

**DOI:** 10.1186/s12957-021-02384-2

**Published:** 2021-09-16

**Authors:** Shiyan Li, Fengjuan Han, Na Qi, Liyang Wen, Jia Li, Cong Feng, Qingling Wang

**Affiliations:** 1 Department of Gynecology, Heilongjiang University of Traditional Chinese Medicine, Harbin, PR China; 2 Department of Acupuncture and Moxibustion, Heilongjiang University of Traditional Chinese Medicine, Harbin, P.R. China; 3 Department of Gynecology, Shenzhen Nanshan Maternal and Child Health Care Hospital, Shenzhen, P.R. China

**Keywords:** Cervical cancer, DEGs, WGCNA, LASSO, Prognosis

## Abstract

**Aim:**

This study aimed to establish a risk model of hub genes to evaluate the prognosis of patients with cervical cancer.

**Methods:**

Based on TCGA and GTEx databases, the differentially expressed genes (DEGs) were screened and then analyzed using GO and KEGG analyses. The weighted gene co-expression network (WGCNA) was then used to perform modular analysis of DEGs. Univariate Cox regression analysis combined with LASSO and Cox-pH was used to select the prognostic genes. Then, multivariate Cox regression analysis was used to screen the hub genes. The risk model was established based on hub genes and evaluated by risk curve, survival state, Kaplan-Meier curve, and receiver operating characteristic (ROC) curve.

**Results:**

We screened 1265 DEGs between cervical cancer and normal samples, of which 620 were downregulated and 645 were upregulated. GO and KEGG analyses revealed that most of the upregulated genes were related to the metastasis of cancer cells, while the downregulated genes mostly acted on the cell cycle. Then, WGCNA mined six modules (red, blue, green, brown, yellow, and gray), and the brown module with the most DEGs and related to multiple cancers was selected for the follow-up study. Eight genes were identified by univariate Cox regression analysis combined with the LASSO Cox-pH model. Then, six hub genes (SLC25A5, ENO1, ANLN, RIBC2, PTTG1, and MCM5) were screened by multivariate Cox regression analysis, and SLC25A5, ANLN, RIBC2, and PTTG1 could be used as independent prognostic factors. Finally, we determined that the risk model established by the six hub genes was effective and stable.

**Conclusions:**

This study supplies the prognostic value of the risk model and the new promising targets for the cervical cancer treatment, and their biological functions need to be further explored.

## Introduction

Cervical cancer is one of the most common tumors in gynecology with high incidence and mortality rate, ranking fourth in the world for common female malignancies [1, 2]. The prognosis of patients with early cervical cancer is favorable, and the 5-year survival rate can reach 90%, while the survival rate of advanced patients, especially those with distant metastasis or recurrence, is significantly decreased [3, 4]. Clinically, traditional treatment methods for cervical cancer still have certain drawbacks. For example, surgical treatment is only suitable for early-stage patients, and postoperative radiotherapy will cause irreversible damage to the ovaries and uterus [5, 6]. Hence, emerging therapies such as targeted therapy and immunotherapy have become a hot spot in cervical cancer research [7], and new diagnostic molecular markers and therapeutic targets are still to be discovered.

The identification of tumor-related genes is an important basis for the study of tumor pathogenesis and the formulation of prevention and treatment measures [8]. Understanding cervical cancer at the gene level provides important clues for etiology research, early diagnosis, and treatment [9]. During the last two decades, the advances of markers screened by high throughput genome sequencing and bioinformatics analysis received more and more attention [10]. Bioinformatics analysis based on gene chip is a common method to detect the difference of RNA expression between different samples on a high-throughput platform, which can screen out differentially expressed genes (DEGs) in a large amount of data [11–13]. Analyses of the microarray data from the public databases, such as The Cancer Genome Atlas (TCGA) database, would provide valuable clues for the investigation of various diseases [14]. The phenotypic variation of complex life is often not caused by a single gene, but by the interaction of multiple genes in the gene network [15]. Therefore, the analysis of gene function, pathway and interaction is the basis of molecular targeted therapy.

Weighted gene co-expression network analysis (WGCNA) can be used to find clusters (modules) of highly related genes, and associate modules with external sample features, thus to identifying hub genes that can be used as therapeutic targets [16]. Until now, WGCNA has been successfully applied in various biological fields, including cancer [17–19]. Horvath et al. found the key pathogenic gene ASPM for glioblastoma using WGCNA, which has become a key gene for related treatment [20]. However, there is still no study on identifying key nodules and genes in cervical cancer using the WGCNA method. Herein, we used WGCNA for the first time to identify key modules related to cervical cancer. Based on the genes contained in these key modules, we obtained a set of genes as prognostic biomarkers using univariate Cox regression analysis, in combination with Cox proportional hazard (PH) model based on LASSO estimation. We expected to provide more powerful biomarkers for the diagnosis and prognosis of patients with cervical cancer.

## Materials and methods

### Data resource

The gene expression information of cervical cancer was downloaded from TCGA and GTEx databases. Ten normal samples were obtained from the GTEx database as well as 3 normal samples and 306 tumor samples were obtained from the TCGA database. Totally, 13 normal samples and 306 tumor samples were obtained from the two databases for subsequent analysis.

### Screening for DEGs

Thirteen normal samples and 306 tumor samples offered from TCGA and GTEx databases were analyzed for DEGs. We used the limma package in R to obtain differentially expressed genes with FDR < 0.05 and |logFC| > 1 as screening conditions. The final results are represented by a volcano map and heat map.

### GO function annotation and KEGG enrichment analysis

GO function annotation was carried out for the differentially expressed genes in Database for Annotation Visualization and Integrated Discovery (DAVID) (https://david.ncifcrf.gov/), including biological process (BP), cell composition (CC), and molecular function (MF). Simultaneously, KEGG function enrichment analysis was carried out to determine the main biological functions and pathways affected by the differentially expressed genes. *P* < 0.05 indicated that the difference was statistically significant.

### WGCNA

WGCNA package (version 1.61) was used to identify the cervical cancer-related modules according to the previous description [21]. In brief, scale-free topology criterion was used to calculate the soft threshold. The optimal soft threshold was selected and the minimum module size was set as 30 genes. The modules were identified using the dynamic tree cut, and the MEDissThres parameter was set to 0.25. After the modules were correlated with clinical features, the modules with Pearson correlation coefficient > 0.75 were selected and merged to obtain the target module.

### Construction of prognostic model

Univariable Cox regression analysis was used to screen out the significant genes. Then, the most useful prognostic marker was selected using Lasso regression analysis, and tenfold cross-validation was utilized to determine the optimal value of penalty parameter λ. The Cox-PH model was used to validate the identified prognosis-related genes. Subsequently, a Multivariate Cox regression model was used to determine the hub genes and independent prognostic factors. The risk curve and survival state of the risk model established by the prognosis-related genes were analyzed. In addition, Kaplan-Meier analysis and receiver operating characteristic (ROC) curve analysis were performed for further verification.

### Statistical analysis

All statistical tests were bilateral, and statistical analyses in this work were performed using R software and SPSS 22.0 (IBM, Armonk, NY, USA). The comparisons were conducted with *T* test, and *P* < 0.05 was regards as significant statistically.

## Results

### Screening DEGs

**Fig. 1:**
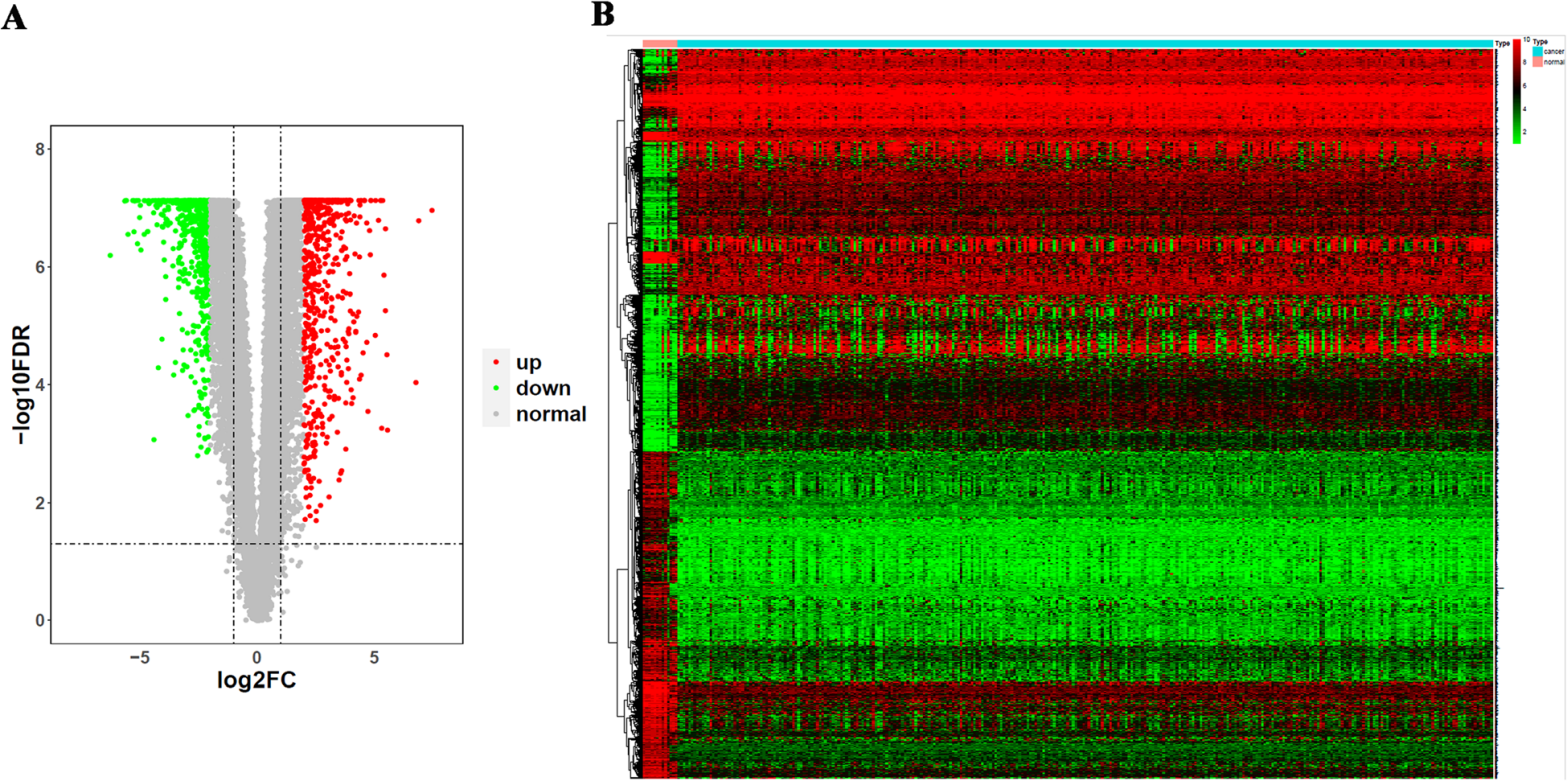
The volcano plot and heat map of differentially expressed genes (DEGs) in cervical cancer samples and controls. **A** The volcano plot for DEGs. Gray dots represented genes with no differential expression, green dots represented downregulated genes, and red dots represented upregulated genes. **B** The heat map for DEGs

Based on the TCGA and GTEx databases, 1265 differentially expressed genes were obtained using the limma package in R with FDR < 0.05 and |logFC| > 1, of which 620 were downregulated and 645 were upregulated in cervical cancer (Fig. 1A, B).

**Fig. 2:**
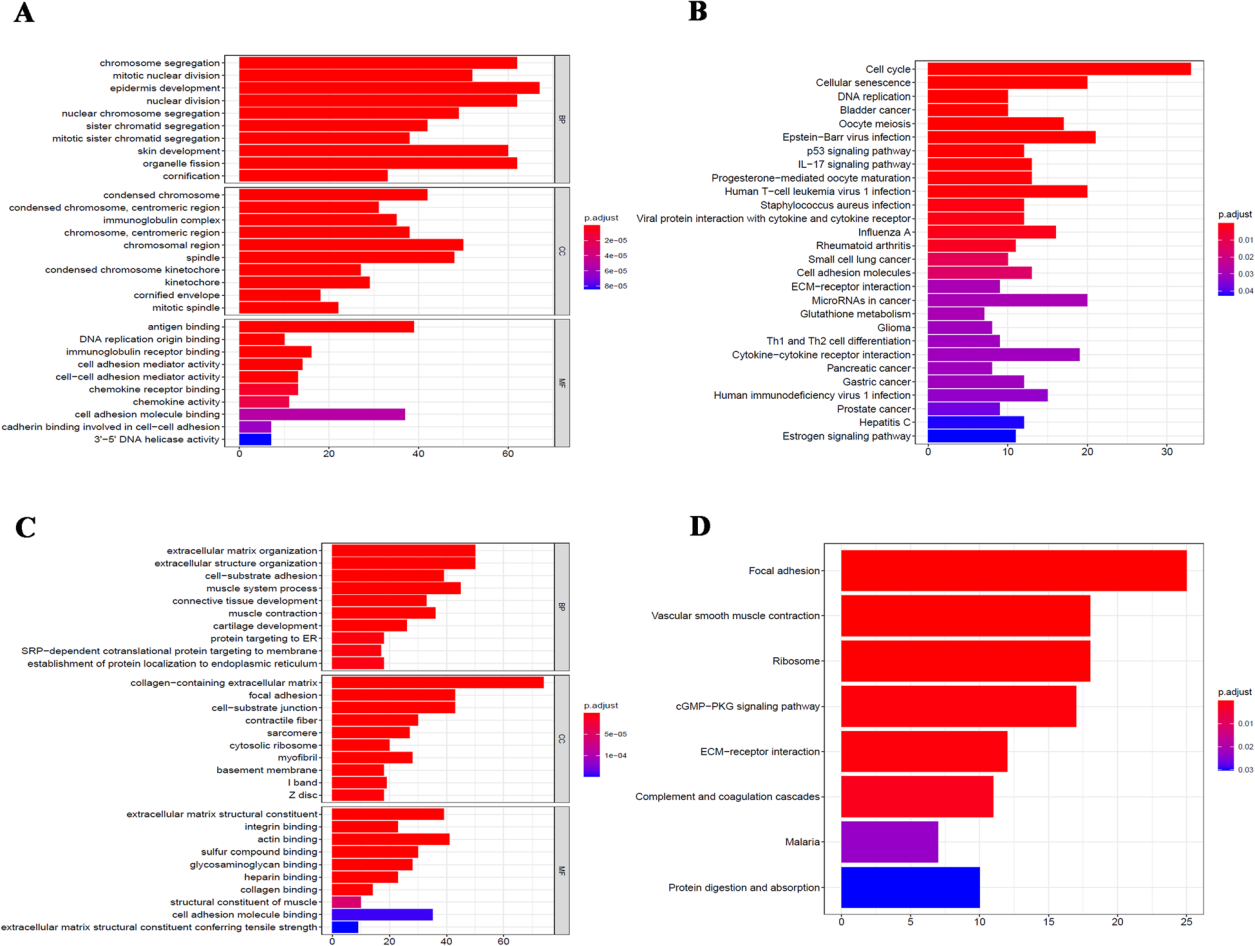
GO and KEGG analyses of differentially expressed genes (DEGs) in cervical cancer samples. **A**, **B** GO and KEGG analysis for downregulated genes. **C**, **D** GO and KEGG analysis for upregulated genes

Then, we used GO and KEGG enrichment to analyze the upregulated and downregulated DEGs respectively. Herein, Fig. 2A, B presented the enrichment of downregulated genes. For GO-BP analysis, downregulated DEGs were markedly enriched in mitosis chromosomes segregation, sister chromatid segregation, and organelle fission. These are all related to the cell cycle and sex cell generation. GO-CC analysis indicated that DEGs are mainly concentrated in chromosomal region and immunoglobulin complex, which are also related to cell cycle and immunity. The mainly enriched MF terms are antigen binding, immunoglobulin receptor binding, and cell-cell adhesion, which are all about regulating the immune and cellular interactions. In addition, KEGG analysis revealed that the top significantly enriched pathway for DEGs is the cell cycle. Combined with GO analysis, most of the downregulated genes act on the cell cycle. Hence, we speculated that after the downregulation of these genes, the cell cycle may be disordered. Meanwhile, the results of upregulated genes were displayed in Fig. 2C, D. GO-BP analysis indicated that the top three enriched GO terms were extracellular matrix organization, extracellular structure organization, and cell-substrate adhesion, which are associated with the occurrence and metastasis of cancer. The results of GO-CC analysis were similar to GO-BP analysis that the upregulated DEGs were mainly enriched in collage-containing extracellular matrix and focal adhesion. Also, for GO-MF analysis, upregulated DEGs were mainly enriched in extracellular matrix structural constituents. Moreover, KEGG analysis indicated that the markedly enriched pathways for DEGs are focal adhesion, vascular smooth muscle contraction, ribosome, and extracellular matrix receptor responses. It follows that most of the upregulated DEGs are related to the changes of internal environment as well as the focus and metastasis of cancer cells.

### WGCNA network module mining

**Fig. 3:**
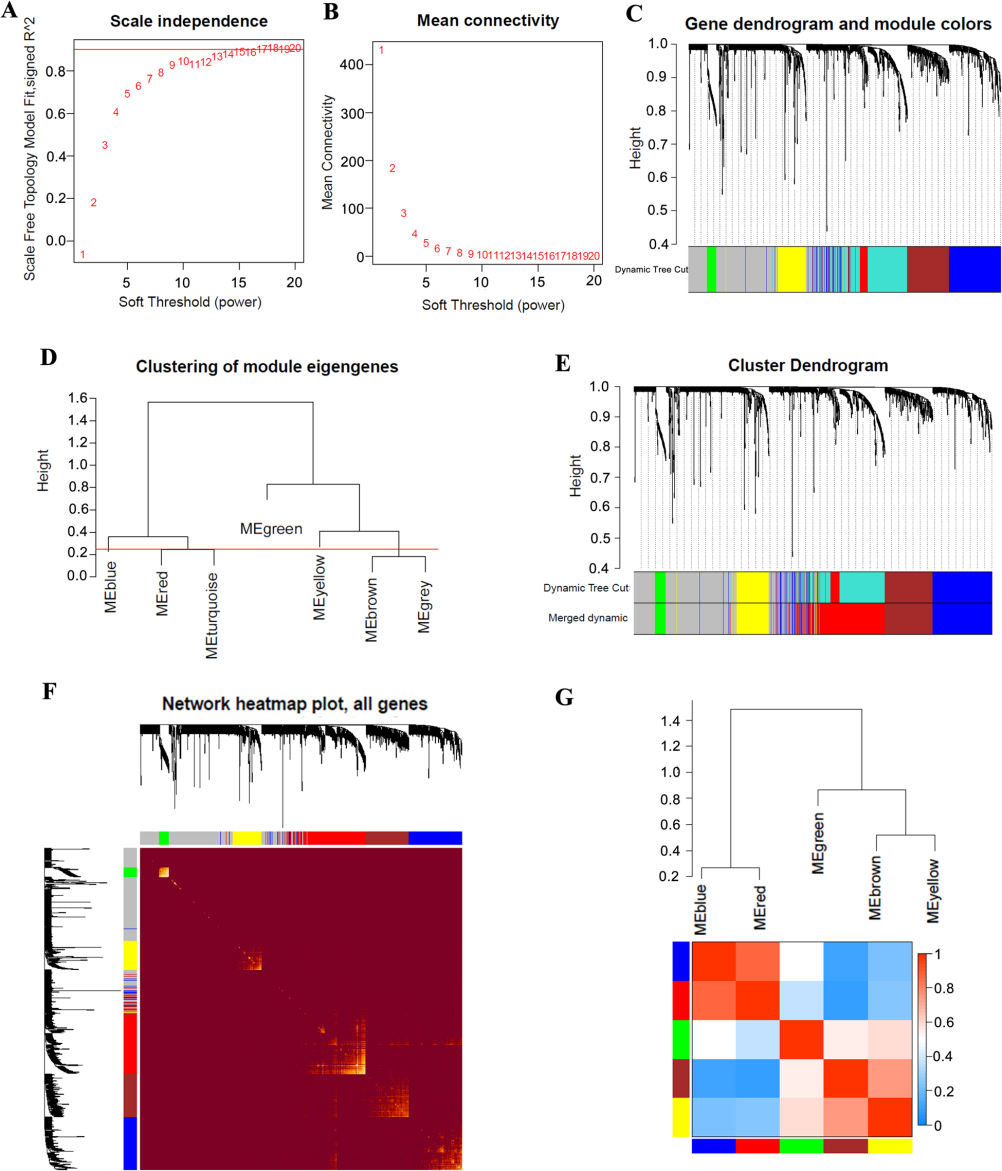
WGCNA network module mining. **A**, **B** Determine the best soft threshold using network topology analysis. When *β* = 13, it satisfies the scale-free topology threshold of 0.9. **C** The gene dendrogram and nodule color of WGCNA. **D** Hierarchical clustering analysis of WGCNA modules. **E** The gene dendrogram is based on clustering. **F** Network heatmap plot of all genes. **G** The correlation between modules

The DEGs screened from TCGA and GTEX databases were applied for WGCNA. Firstly, we calculate the soft threshold using scale-free topology criterion. The connectivity between genes in the gene network satisfied the scale-free network distribution when soft threshold power of *β* = 13 (scale-free *R*^2^ = 0.9) (Fig. 3A, B). Then, co-expression modules (cut height, ≥0.25) were mined using the phylogenetic tree (Fig. 3C). The modules were analyzed by hierarchical clustering, and the modules on the same branch showed similar gene expression patterns (Fig. 3D). Similar gene modules are then identified and combined, and then 6 co-expression modules (red, blue, green, brown, yellow, and gray) were obtained (Fig. 3E). Subsequently, the gene clustering was visualized (Fig. 3E) and the correlation between modules was analyzed (Fig. 3F). All data revealed that the higher correlation was presented between the blue and red modules as well as the brown and yellow modules.

**Fig. 4:**
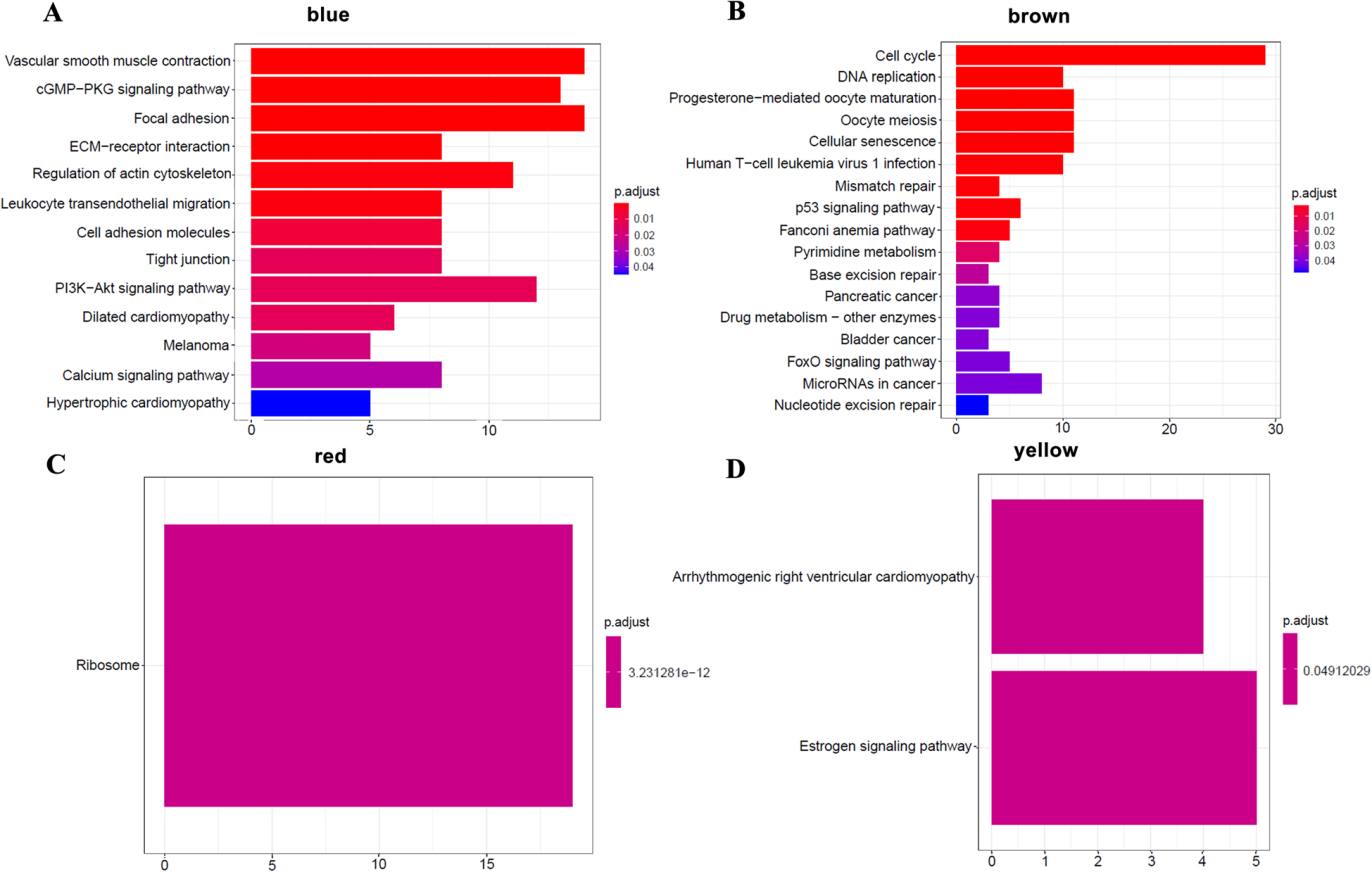
The most DEGs were enriched in the brown module. KEGG analysis for DEGs in **A** blue, **B** brown, **C** red, and **D** yellow modules

Additionally, KEGG enrichment analysis was performed for each module (Fig. 4). The genes in the brown module (Fig. 4B) are mainly involved in the cell cycle, DNA replication, and signaling pathways and are associated with multiple cancers. In addition, the brown module has the highest concentration, so we chose the brown module as the research object.

### Establishment of prognostic model based on independent prognostic gene

**Fig. 5:**
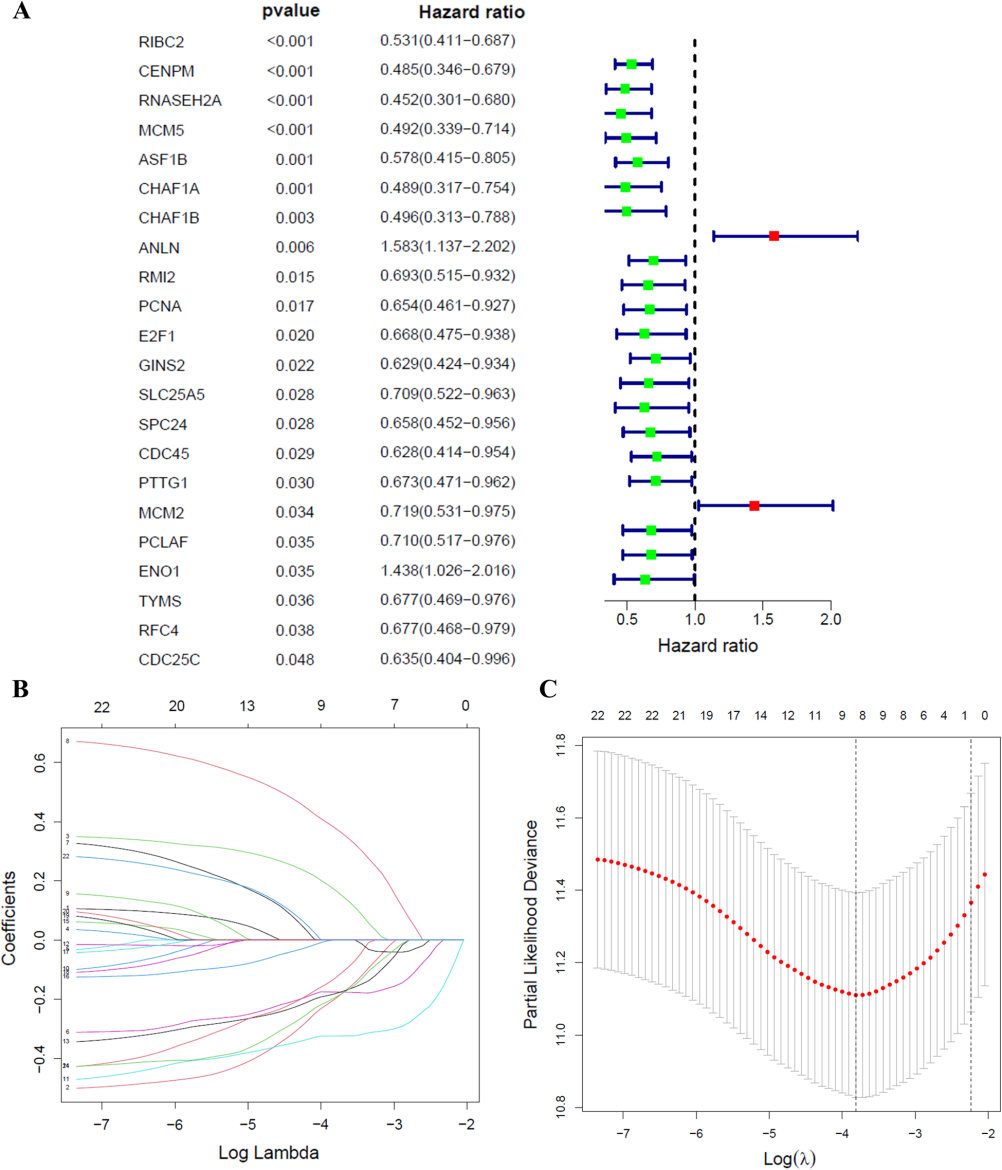
Identification and screening of the prognosis-related genes. **A** Univariate Cox regression analysis was used to assess the genes that related to prognosis. **B** Tenfold cross-validation for tuning parameter selection in the LASSO model. **(C)** LASSO coefficient profiles of 22 prognostic DEGs for cervical cancer

**Fig. 6:**
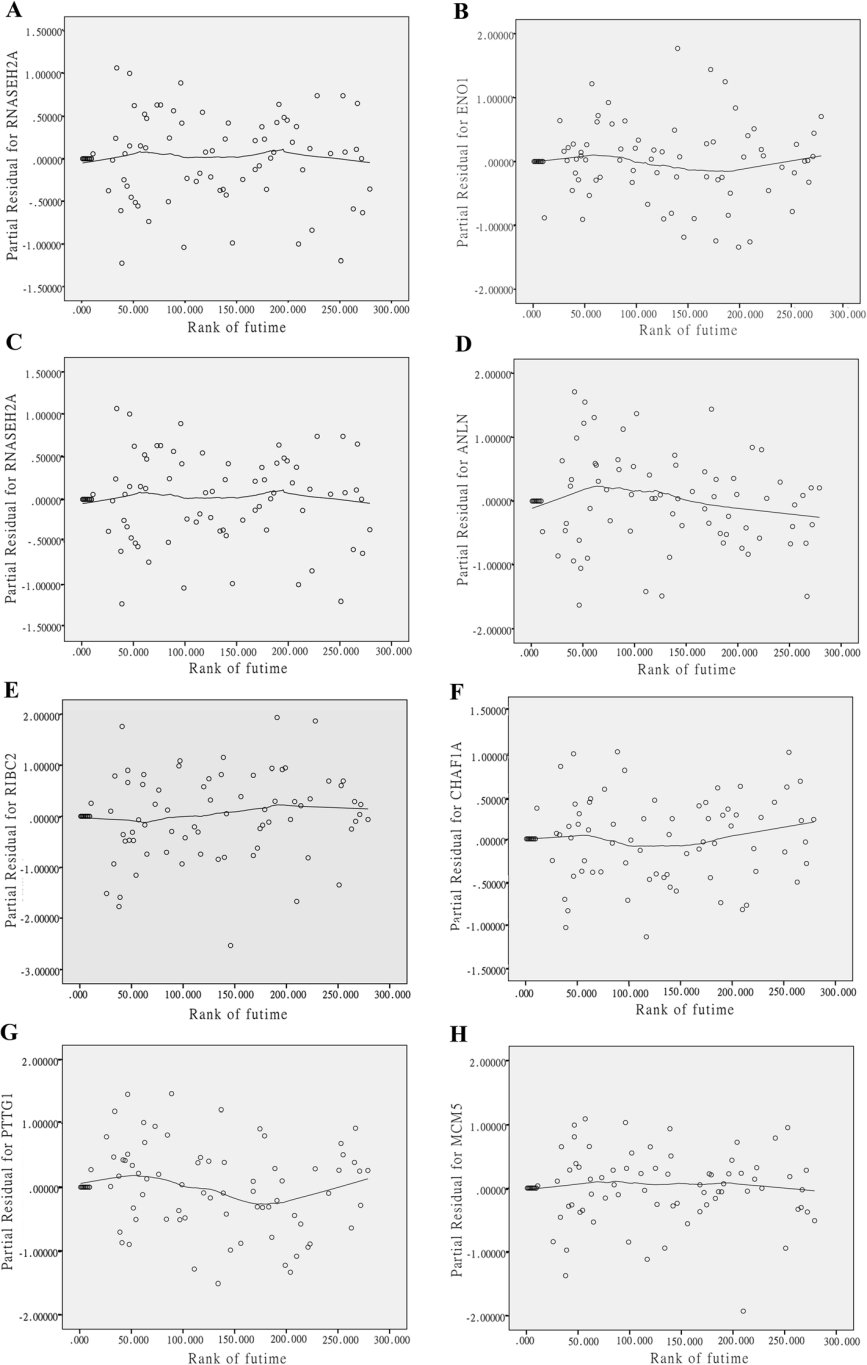
The risk ratio of gene expression was independent of survival time. PH (proportional hazards) test for **A** SLC25A5, **B** ENO1, **C** RNASEH2A, **D** ANLN, **E** RIBC2, **F** CHAF1A, **G** PTTG1, and **H** MCM5

Univariate cox regression analysis was performed to obtain the meaningful genes in brown module. As presented in Fig. 5A, 22 genes were considered to be associated with prognosis. The immunization scoring model of the training cohort was established using LASSO Cox regression analysis (Fig. 5B, C). Eight prognostic genes (SLC25A5, ENO1, RNASEH2A, ANLN, RIBC2, CHAF1A, PTTG1, and MCM5) were screened and PH (proportional hazards) test was performed on them. As presented in Fig. 6A–H, the risk ratio of gene expression changed little over time and was independent of survival time. Hence, all of the above genes can be used for risk regression analysis and modeling.

**Fig. 7:**
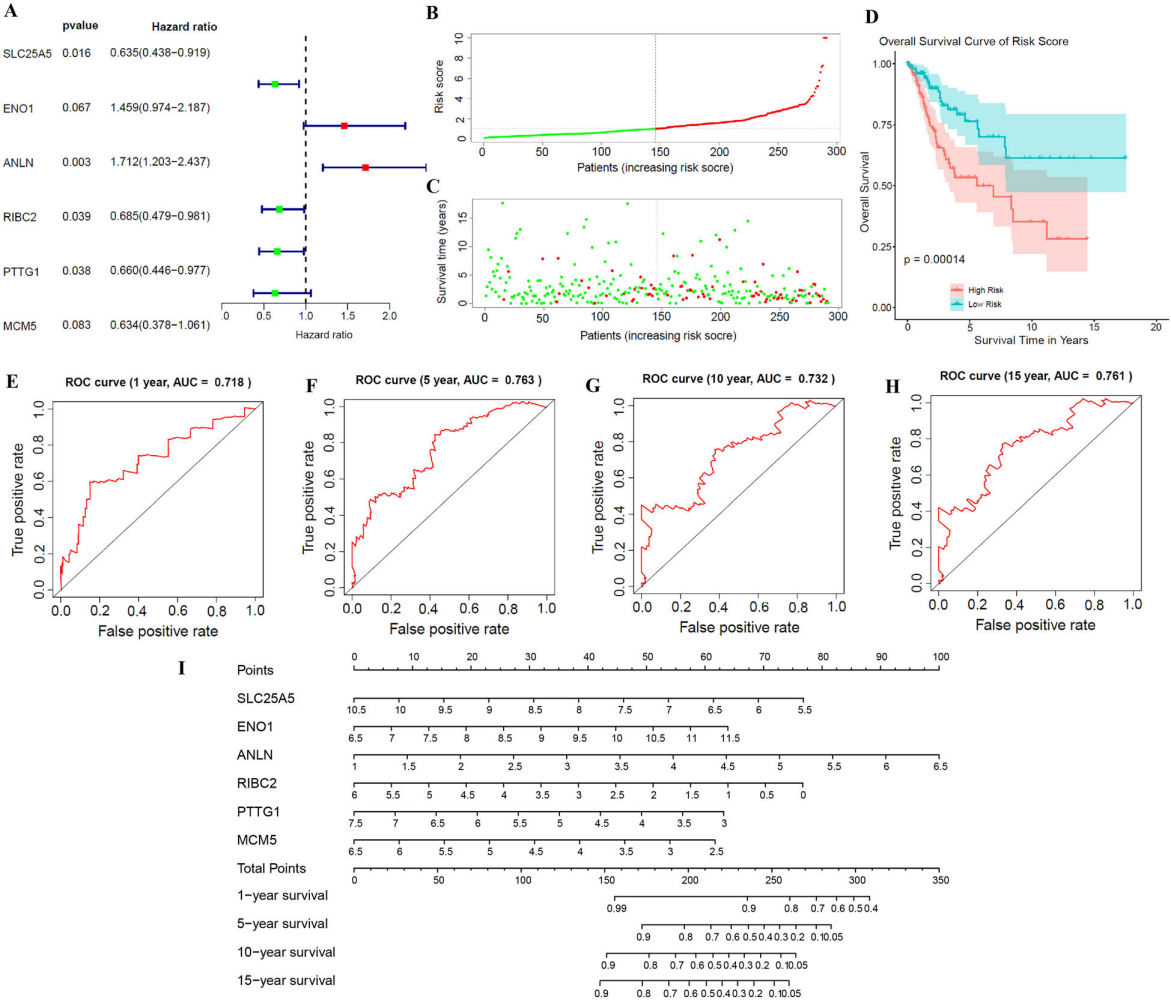
Establish and verify the risk model for cervical cancer. **A** Multivariate Cox regression analysis for the prognosis-related genes. **B** Risk curve and **C** survival state of risk model based on six genes (SLC25A5, ENO1, ANLN, RIBC2, PTTG1, and MCM5). **D** KM curve based on the risk model. ROC curve based on the risk model at **E** 1 year, **F** 5 years, **G** 10 years, and **H** 15 years. **I** Nomogram was established to predict the risk score and survival probability of patients with cervical cancer

Then, a risk model with six genes (SLC25A5, ENO1, ANLN, RIBC2, PTTG1, and MCM5) as influencing factors was obtained by multivariate Cox regression analysis (Fig. 7A). Wherein, the *P* values of SLC25A5, ANLN, RIBC2, and PTTG1 were all less than 0.05 and could be used as independent prognostic factors. Furthermore, we assessed the risk model with six hub genes. First we analyzed the risk curve and survival state. As shown in Fig. 7B, C, the high risk group presented more deaths and a lower average number of years of survival. Then, we plotted the KM curve and the time-dependent ROC curve. The KM curve indicated that the survival rate of the high-risk group was significantly lower than that of the low-risk group (Fig. 7D). Meanwhile, the ROC curve revealed that 1 year, 5 years, 10 years and 15 years survival rate can be all predicted by using the risk model (Fig. 7E–H). Also, nomogram indicated that the total score of nomogram was helpful to provide a quantitative method for predicting the prognosis of cervical cancer (Fig. 7I). These data illustrated that six genes (SLC25A5, ENO1, ANLN, RIBC2, PTTG1, and MCM5) can be used to establish the risk model, and the model was stable.

## Discussion

For the moment, targeted therapy has become a hot spot in the treatment of cervical cancer [22]. Several biomarkers for cervical cancer have been identified in recent years. For example, Heng Zou et al. reported a novel circ_0018289/miR-183-5p/TMED5 regulatory network as a novel molecular basis underlying the modulation of cervical cancer [23]. Studies provided evidence that targeting IGF1 by miR-186-3p can regulate cervical cancer progression, and miR-125 inhibited cervical cancer progression and development by inhibiting VEGF and PI3K/AKT signaling pathway, providing more insights into the treatment of cervical cancer [2, 24]. However, the biomarkers of targeted therapy for cervical cancer are complex and diverse, which also need to be fully excavated and explored [25]. In this work, we found 1265 DEGs between cervical cancer and normal samples, of which 620 were downregulated and 645 were upregulated. The upregulated DEGs were mainly related to tumor cell metastasis, while the downregulated genes were mainly related to the cell cycle. Moreover, immune cells can successfully distinguish tumor tissue from normal tissue. We excavated six modules using WGCNA, and then analyzed the brown module with the most DEGs and related to multiple cancers. The hub genes such as SLC25A5, ENO1, ANLN, RIBC2, PTTG1, and MCM5 can be used to establish the risk model for CC, and the model was stable. Furthermore, SLC25A5, ANLN, RIBC2, and PTTG1 could be used as independent prognostic factors.

Increasing evidence demonstrated that genomic instability, oncogene activation, aberrant expression of important genes, and alterations of signaling pathways are vital factors and contribute to cancer pathogenesis [26]. WGCNA has been widely utilized for identifying gene modules associated with several diseases and screening potential therapeutic targets [10]. WGCNA clusters highly related genes into different modules to construct co-expression modules, and then analyzes the functions of these modules [27]. For example, KEGG is used to analyze the pathways associated with DEGs in these modules [27]. By WGCNA algorithm, Wenxi Yan et al. identified 5 significantly stable gene modules related to colon cancer and constructed a 12-gene prognostic model via further bioinformatics analysis [10]. In this work, we identified six modules using WGCNA. We found that the genes of the brown module mainly act on the cell cycle, DNA replication and are associated with multiple cancers. In order to identify the prognostic genes in the brown module, we performed univariate Cox-Lasso regression analysis, and finally screened eight genes. Then, six prognostic hub genes, SLC25A5, ENO1, ANLN, RIBC2, PTTG1, and MCM5, were identified by multivariate Cox regression analysis.

SLC25A5 is a by-product of ninucleotide transferase, encoding the transporter ADP/ATP [28]. SLC25A5 deletion can lead to mitochondrial dysfunction and oxidative stress. Hence, the development of B cells may be related to SLC25A5 [29]. Moreover, SLC25A5 is considered to be associated with lymph node metastasis of hepatocellular carcinoma [30]. ENO1 is expressed on the surface of tumor cells and promoted tumor cells invasion, which has diagnostic and prognostic value in many cancers [31]. ANLN, a protein coding gene, is highly expressed in many cancers [32]. Upregulation of ANLN leads to shorter survival time in patients with colorectal cancer [33] and breast cancer [34]. Importantly, Leilei Xia et al. found that ANLN is a prognostic factor for cervical cancer [35]. RIBC2 is abnormally expressed in renal clear cell carcinoma, breast cancer, and ovarian serous cystadenocarcinoma [36]. Importantly, RIBC2 has been identified as a key gene related to the progression and prognosis of cervical squamous cell carcinoma [37]. PTTG1, an oncogene, has the ability to regulate the self-renewal and epithelial mesenchymal transformation of cancer stem cells [38]. In terms of biological function, PTTG1 is regulated by various lncRNAs and miRNAs and plays a role in the malignant progression of cervical cancer [39–41]. MCM5, a minichromosome maintenance protein, is upregulated in cervical cancer and promoted cell proliferation. Also, MCM5 is closely related to clinical stage, lymph node metastasis, distant metastasis, and histological grade [42]. In this paper, we used these six genes to establish an effective and stable risk model for the prognosis of cervical cancer.

## Conclusions

In summing, we screened six prognostic hub genes by WGCNA combined with LASSO Cox-PH analysis and established a risk model. We also proved that the risk model is effective and stable. Our results provide a new strategy for targeted therapy of cervical cancer. However, in vitro and in vivo biological function experiments are needed to verify our results.

## Data Availability

The data that support the findings of this study are available from the corresponding author upon reasonable request.
